# Announcement of new journal and call for submission of papers: *Surgical Neurology International*

**DOI:** 10.4103/2152-7806.62259

**Published:** 2010-04-07

**Authors:** James I. Ausman

**Affiliations:** Editor-in-Chief, Surgical Neurology International, USA

I am pleased to announce the formation of *Surgical* *Neurology International* (SNI), a new OPEN ACCESS, Internet-only journal, accessible to all neurosurgeons. OPEN ACCESS means that anyone in the world with access to the Internet can visit the website, view the journal, obtain its papers, its editorials, and comments for FREE!

*Surgical Neurology International* will serve approximately 35,000 neurosurgeons around the world. It will potentially have the largest circulation of any neurosurgical journal. *SNI* will have the same editorial policy and principally the same Editorial Board as I established and upheld as Editor-in-Chief of *Surgical Neurology* for 15 years. *SNI* will accept papers on clinical neurosurgery and other clinical and basic neurosciences with topics of interest to our audience. It will have editorial comments about controversies, the principles of medical and neurosurgical practice, socio-economics, politics, ethics, and science, as were expressed in the past.

In addition to accepting research papers from all over the world, *SNI* will have an advanced educational website for residents and practicing neurosurgeons, with content provided by the UCLA Department of Neurosurgery and its guest lecturers under the direction of Nestor Gonzalez and with the cooperation of Neil Martin, the Department Chairman. This content, as well as downloads of papers, will be available at no charge. For those neurosurgeons who cannot afford a journal, cannot access one easily, or cannot go to meetings, *Surgical Neurology International* will be their source of information.

*Surgical Neurology International* wants to involve neurosurgeons, particularly the young neurosurgeons who have a difficult time for gaining recognition in many countries around the world. *SNI* will have new and innovative Clinical Decision Support sites for real time use by neurosurgeons in clinical cases they see every day. *SNI* will also have the latest social networking sites and individual communication options so everyone can access the journal anywhere, any time. It will be twenty-first century education.

For those who are interested, they can receive notices of new papers being published each month. When papers are accepted, they will be published within days rather than weeks or months. All papers will be searchable through PubMed Central, a division of PubMed, and will be searchable on PubMed and other commonly-used databases.

The only cost will be to authors of accepted papers. This fee will be US$400 to publish a paper after it is accepted. For short communications and case reports, the cost will be US$180. We are working on ways to eliminate that fee by obtaining advertising or grants. There is no additional charge for color pictures or videos. For those who cannot afford this author's cost, it can be waived by decision of the Editor.

Our publisher is Medknow Publishers in Mumbai, India. We thank Atul Goel for directing us to this organization that publishes many OPEN ACCESS medical journals. I also want to thank Bob Goodkin, Pat Kelly, Ben Roitberg, Konstantin Slavin, Neil Martin, and Nestor Gonzales for their support in bringing this new journal to all neurosurgeons in the world.

You can access our new Web site at www.surgicalneurologyint.com. This site includes a link for authors to submit their new manuscripts, and is also the Web site to use to access the educational content, which will be operational soon. This Web site will have other features we are developing to help you exchange information with your colleagues about challenging cases, an imaging site for interesting cases, chat rooms, etc. If you would like to receive Table of Contents through e-mails or RSS alerts, please access the Web site and follow the instructions under “Staying in touch with the journal.”

If you are interested in becoming involved with the journal, *SNI*, in any of its aspects, please let me know at jamesausman@mac.com.

We hope that neurosurgeons everywhere will enjoy this new publication. This is your journal, and we are open to any ideas you have to make it beneficial to you and to your practice. We would be delighted if you would share this information with your colleagues.

